# Multiscale structural mapping of Alzheimer’s disease neurodegeneration

**DOI:** 10.1016/j.nicl.2022.102948

**Published:** 2022-01-22

**Authors:** Ikbeom Jang, Binyin Li, Joost M. Riphagen, Bradford C. Dickerson, David H. Salat

**Affiliations:** a Athinoula A. Martinos Center for Biomedical Imaging, Massachusetts General Hospital, Charlestown, MA, USA; b Department of Radiology, Massachusetts General Hospital, Harvard Medical School, Boston, MA, USA; c Department of Neurology, Ruijin Hospital, Shanghai Jiao Tong University School of Medicine, Shanghai, China; d Faculty of Health, Medicine and Life Sciences, School for Mental Health and Neuroscience, Alzheimer Centre Limburg, Maastricht University, Maastricht, the Netherlands; e Department of Neurology, Massachusetts General Hospital, Harvard Medical School, Boston, MA, USA; f Department of Psychiatry, Massachusetts General Hospital, Harvard Medical School, Boston, MA, USA; g Neuroimaging Research for Veterans Center, VA Boston Healthcare System, Boston, MA, USA

**Keywords:** Alzheimer’s disease, Mild cognitive impairment, Biomarker, Gray matter to white matter contrast, Multiscale mapping, Cortical thickness

## Abstract

The recently described biological framework of Alzheimer’s disease (AD) emphasizes three types of pathology to characterize this disorder, referred to as the ‘amyloid/tau/neurodegeneration’ (A-T-N) status. The ‘neurodegenerative’ component is typically defined by atrophy measures derived from structural magnetic resonance imaging (MRI) such as hippocampal volume. Neurodegeneration measures from imaging are associated with disease symptoms and prognosis. Thus, sensitive image-based quantification of neurodegeneration in AD has an important role in a range of clinical and research operations. Although hippocampal volume is a sensitive metric of neurodegeneration, this measure is impacted by several clinical conditions other than AD and therefore lacks specificity. In contrast, selective regional cortical atrophy, known as the ‘cortical signature of AD’ provides greater specificity to AD pathology. Although atrophy is apparent even in the preclinical stages of the disease, it is possible that increased sensitivity to degeneration could be achieved by including tissue microstructural properties in the neurodegeneration measure. However, to facilitate clinical feasibility, such information should be obtainable from a single, short, noninvasive imaging protocol. We propose a multiscale MRI procedure that advances prior work through the quantification of features at both macrostructural (morphometry) and microstructural (tissue properties obtained from multiple layers of cortex and subcortical white matter) scales from a single structural brain image (referred to as ‘multi-scale structural mapping’; MSSM). Vertex-wise partial least squares (PLS) regression was used to compress these multi-scale structural features. When contrasting patients with AD to cognitively intact matched older adults, the MSSM procedure showed substantially broader regional group differences including areas that were not statistically significant when using cortical thickness alone. Further, with multiple machine learning algorithms and ensemble procedures, we found that MSSM provides accurate detection of individuals with AD dementia (AUROC = 0.962, AUPRC = 0.976) and individuals with mild cognitive impairment (MCI) that subsequently progressed to AD dementia (AUROC = 0.908, AUPRC = 0.910). The findings demonstrate the critical advancement of neurodegeneration quantification provided through multiscale mapping. Future work will determine the sensitivity of this technique for accurately detecting individuals with earlier impairment and biomarker positivity in the absence of impairment.

## Highlights

• A multiscale structural mapping (MSSM) procedure is proposed for the quantification of neurodegeneration in Alzheimer's disease using a single structural brain image.
• The MSSM procedure captures both macrostructural properties and indirect index of tissue microstructure throughout the cerebral cortex.
• The MSSM procedure provides enhanced ability for the detection of degeneration in Alzheimer’s disease and mild cognitive impairment compared to traditional measures such as cortical thickness and hippocampal volume and therefore may provide a sensitive measure of Alzheimer’s disease neurodegeneration.

## Graphical abstract

**Figure:**
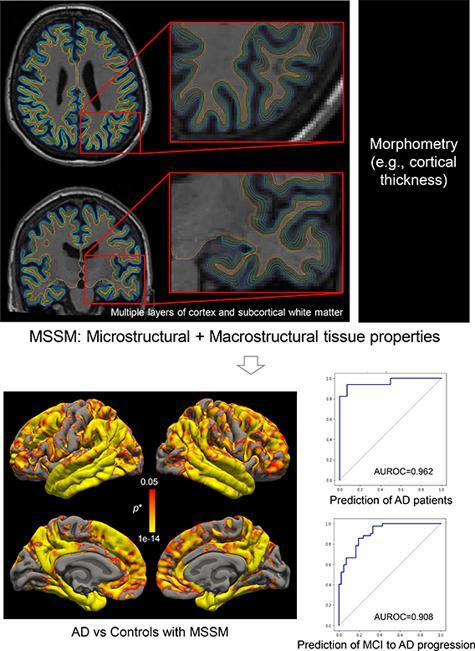

## 1 Introduction

One in three seniors dies with dementia, and Alzheimer’s disease (AD) is the most common cause of dementia in older adults in the U.S. (Alzheimer’s Association., 2021) AD begins potentially a decade or more prior to the time when clinical symptoms are apparent (Jack et al., 2013, Bateman et al., 2012, Jack et al., 2010, Buchhave et al., 2012, Jack et al., 2011, Caroli and Frisoni, 2010, Jack et al., 2012, Förster et al., 2012, Landau et al., 2012), therefore, clinical symptomatic identification of AD is relatively late in the pathologic process and at a time when novel therapeutics will be less effective. Recent work aims to detect AD early after pathology is initiated, well in advance of clinical expression of symptoms. A commonly used biological framework for AD, ‘A-T-N’, references brain patterns of amyloid, tau, and neurodegeneration, respectively. The primary validated AD-defining biomarkers, amyloid and tau, can be quantified through lumbar puncture cerebrospinal fluid assay or by positron emission tomography (PET) using radiotracers that target these abnormal proteins. Amyloid and tau can be detected early in AD pathogenesis with these promising techniques, yet these measures are currently limited for routine screening given cost and invasiveness and they are only possible at specialized clinics. Neurodegeneration measures from imaging are associated with disease severity and prognosis (Benvenutto et al., 2018, Brickman et al., 2018, Aziz et al., 2017), and MRI is an alternative tool to assess AD neurodegeneration, which is safe, non-invasive, and more accessible in clinical settings. Advances in brain imaging with machine learning techniques made it possible to identify individuals with AD dementia or mild cognitive impairment (MCI) (Belathur Suresh et al., 2018, Cho et al., 2012, Suk et al., 2014, Sarraf et al., 2019, Bloch and Friedrich, 2021, Lin et al., 2018, Li et al., 2021, Guo et al., 2017, Ye et al., 2012, Gao et al., 2020, Shi et al., 2018, Liu et al., 2014, Wolz et al., 2011, Lu et al., 2018, Sørensen et al., 2016, Popuri et al., 2020, Sun et al., 2017, Tong et al., 2017, Allison et al., 2019, Noor et al., 2019, Choi et al., 2020, Zhang et al., 2011, Westman et al., 2012, Park et al., 2017, Davatzikos et al., 2008, Desikan et al., 2009, Sørensen et al., 2017, Zhu et al., 2017, McEvoy et al., 2009, Magnin et al., 2009, Mattsson et al., 2019, Janghel and Rathore, 2021) using fairly routine structural MRI procedures and therefore MRI provides an optimal ‘first pass’ screen of patients. A simple metric such as hippocampal volume is sensitive to AD neurodegeneration; however hippocampal volume is impacted by a range of conditions and is not specific to AD. Alternatively, our group and others have demonstrated that high-resolution structural MRI can be used for detection and quantification of highly specific patterns of cortical neurodegeneration in patients and that these measures are associated with symptoms and prognosis, (Cho et al., 2012, Wolz et al., 2011, Salat, 2004, Salat et al., 2009, Dickerson et al., 2009, Bakkour et al., 2013). For example, we used computer models of cortical morphometry to quantify features selected from AD ‘cortical signature’ regions (Dickerson et al., 2009) to predict patients with AD through simple support vector machine (SVM) classifiers (Belathur Suresh et al., 2018).

Although common morphometric procedures such as cortical thickness and gray matter volumes are typically utilized in structural MRI studies, novel microstructural properties are also quantifiable from a standard structural T1 image. For example, we have demonstrated that tissue signal properties such as gray matter (GM)/white matter (WM) contrast are altered with aging (Salat, 2004) and Alzheimer’s disease (Salat et al., 2009) and are useful in detecting MCI who progress to dementia (Jefferson et al., 2015). We propose to extend this prior work through the implementation of a novel multiscale MRI procedure allowing quantification of features at both macrostructural (standard morphometry such as cortical thickness) and microstructural scales from a single T1-weighted structural MR image to quantify brain tissue integrity across multiple spatial scales (referred to as ‘multi-scale structural mapping’; MSSM). The procedure is highly sensitive and could be clinically feasible in future implementations.

We examined 1) the added value of the MSSM features over traditional morphometry measured by cortical thickness and hippocampal volume, and 2) whether the procedure could be used to identify AD patients and to predict MCI to AD conversion. We find that the MSSM procedure is more sensitive to regional neurodegeneration than cortical thickness alone and is effective in the classification of patients with AD dementia as well as individuals with MCI who subsequently progress to AD dementia. These new highly sensitive and specific procedures may have applications in early screening for enhancing diagnostics.

## 2 Methods

### 2.1 Participants

Images were obtained from the Alzheimer’s Disease Neuroimaging Initiative (ADNI)-2/GO database (https://adni.loni.usc.edu) for 239 participants. There were 65 participants who were diagnosed with AD dementia (age = 75.3 ± 8.3) and 90 age/sex/education-matched controls who are cognitively normal (CN; age = 65–85). 84 were diagnosed for MCI (age = 71.4 ± 7.0). Half of the MCI individuals progressed to AD dementia within the next 3 years (MCI-C; age = 72.4 ± 7.4) and the other half of the MCI did not convert to AD within 3 years (MCI-NC; age = 70.4 ± 6.5). The MCI-C and MCI-NC groups were age/sex/education-matched. Inclusion criteria for AD and MCI are described in Appendix. Demographics are provided in Table 1. Initial investigations suggested subtle variation in contrast measures across scanner vendors. Since this study is a ‘proof of concept’ to show the effectiveness of the MSSM procedure, we, therefore, limited this initial investigation to a single MRI manufacturer (however, three scanner platforms were represented). Limiting data to a single MRI manufacturer and matching groups for age, sex, and education level left us reduced sample size. We, therefore, tried to keep as many participants as possible and chose not to use amyloid or tau biomarkers for filtering subjects in each group.

**Table 1:** Participant demographics and clinical characteristics. Only data used for the analyses are included in the table.

	AD	Controls		MCI-C	MCI-NC
Number of participants	65	90		42	42
Age (year)	75.3 ± 8.3	74.1 ± 6.5		72.4 ± 7.4	70.4 ± 6.5
Gender (female/male)	24 / 41	33 / 57		21 / 21	21 / 21
Education (year)	16.0 ± 2.5	16.6 ± 2.6		16.6 ± 2.7	16.8 ± 2.2
Proportion of non-Hispanic white	86.2%	84.4%		100.0%	90.5%
CDR-SB	4.5 ± 1.9^**^	0.0 ± 0.1		2.3 ± 1.1^**^	0.9 ± 0.4
MMSE	23.1 ± 2.1^**^	29.1 ± 1.2		27.4 ± 2.1^**^	28.5 ± 1.5
MoCA	16.9 ± 4.4^**^	25.9 ± 2.5		21.0 ± 2.8^**^	24.5 ± 2.6
FAQ	11.8 ± 6.8^**^	0.2 ± 0.7		4.6 ± 4.0^**^	0.8 ± 1.3
RAVLT Immediate	22.6 ± 8.1^**^	46.7 ± 10.5		28.6 ± 8.2^**^	43.1 ± 11.3
RAVLT Percent Forgetting	87.6 ± 23.5^**^	33.2 ± 26.7		77.5 ± 27.2^**^	44.3 ± 33.5
ADAS-Cog 11	20.1 ± 6.8^**^	5.8 ± 3.0		13.8 ± 5.1^**^	7.5 ± 2.7
ADAS-Cog 13	30.3 ± 8.1^**^	8.8 ± 4.6		22.1 ± 6.5^**^	11.3 ± 4.3

Abbreviations: AD, Alzheimer’s disease; MCI-C, MCI who progress to AD dementia; MCI-NC, MCI who do not progress to AD dementia in the timeframe of follow-up; CDR-SB, Clinical Dementia Rating Sum of Boxes; MMSE, Mini-Mental State Exam; MoCA, Montreal Cognitive Assessment; FAQ, Functional Activities Questionnaire; RAVLT, Rey Auditory Verbal Learning Test; RAVLT Immediate, the sum of RAVLT scores from 5 first trials (Trials 1 to 5); RAVLT Percent Forgetting, the score of Trial 5 minus score of the delayed recall then divided by the score of Trial 5; ADAS-Cog 11, Alzheimer's Disease Assessment Scale 11 cognitive items; ADAS-Cog 13, ADAS-Cog 11 plus a delayed recall task and the Digit Symbol Substitution Test. ^**^p < 0.01 for comparisons between AD and controls or between MCI-C and MCI-NC.

### 2.2 MRI acquisition

Brain images were acquired with 3D T1-weighted magnetization-prepared rapid gradient-echo (MP-RAGE) using 3 T MRI scanners. Data were collected from 33 imaging sites that use Siemens Skyra, Verio, or TIM Trio. Imaging followed ADNI-2 protocols and the parameters used are TR = 2300 ms, TE = 2.98 ms, flip angle = 9°, voxel size = 1 × 1 × 1.2 mm^3^ (Bateman et al., 2012). Fully-sampled data from the initial visit were used in the study.

### 2.3 MRI preprocessing

**Fig. 1:**
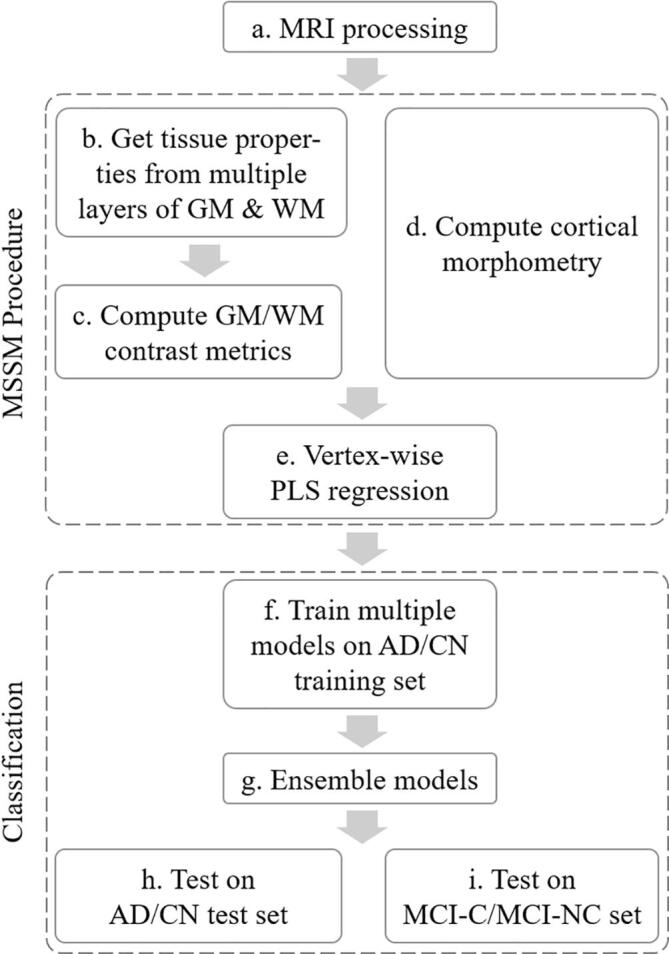
Flowchart of the proposed MSSM procedure and classification. GM, gray matter; WM, white matter; PLS, partial least squares; AD, individuals diagnosed with Alzheimer’s disease; CN, cognitively normal controls; MCI-C, MCI individuals who converted to AD; MCI-NC, MCI individuals who did not convert to AD.

MR images were corrected for gradient non-linearity distortion. Then, the B1 non-uniformity correction procedure was applied to correct non-uniformities in the image intensity. The residual non-uniformities were mitigated using N3 bias field correction (Tustison et al., 2010), which uses histogram peak sharpening, when necessary. Images were warped into a common space for group-level analyses (Fig. 1, panel a).

### 2.4 Multiscale structural mapping (MSSM) procedure

**Fig. 2:**
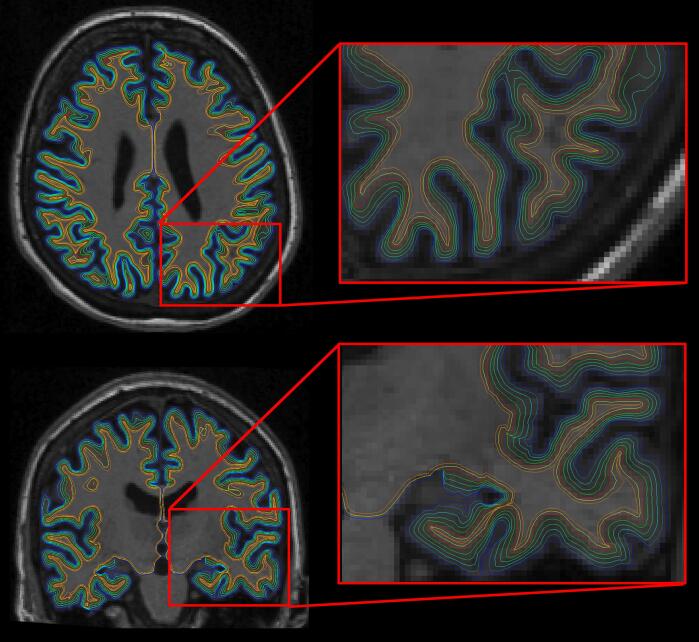
Microstructural feature map generation using a structural T1-weighted image. We expanded the intensity/contrast metrics to include tissue sampling from multiple points through the thickness of the cortical ribbon and subjacent white matter to obtain an array of intensity-linked features. Gray matter intensities were measured at depths of 20%, 40%, 60%, and 80% (surfaces between the blue and red) through the thickness of the cortical ribbon, starting from the gray/white border (red surfaces) towards the gray/CSF border (blue surfaces). White matter intensities were measured 0.5 mm and 1 mm subjacent to the gray/white border (surfaces underneath the red). (For interpretation of the references to color in this figure legend, the reader is referred to the web version of this article.)

Data processing for morphometry and cortical surface mapping followed our prior work and other publications describing these procedures (Salat et al., 2011, Fischl et al., 2004, Fischl et al., 2002, Fischl and Dale, 2000, Fischl et al., 1999, Dale et al., 1999, Ségonne et al., 2004, Fischl et al., 1999) (Fig. 1, panel a). In brief, cortical surface modeling was performed using FreeSurfer image analysis suite v5.3 (http://surfer.nmr.mgh.harvard.edu) (Fischl et al., 2004, Fischl et al., 2002, Fischl and Dale, 2000, Fischl et al., 1999, Dale et al., 1999, Ségonne et al., 2004, Fischl et al., 1999). It provides robust gray matter/white matter segmentation to model the surface of the gray matter/white matter interface as well as the gray matter/cerebrospinal fluid (CSF) interface. Once the cortical models were complete, several deformable procedures were performed for further data processing and analysis including morphometric maps of cortical thickness, curvature, sulcal depth, etc. Cortical gray matter tissue signal properties were obtained by creating a set of additional surfaces in the interior of the cortical ribbon at different depths through the cortical thickness (20%, 40%, 60%, 80%) starting from the gray matter/white matter border and projecting towards the gray matter/cerebrospinal fluid border. Fig. 2 shows an example of this procedure examining the gray/white contrast ratio from multiple surface pairs. White matter intensities were sampled at 5 mm and 1 mm subjacent to the gray/white matter border (Fig. 1, panel b). Ratios of each pair of surfaces were created for a total of 8 contrast features (4 gray matter/2 white matter contrast features) at each cortical surface vertex (Fig. 1, panel c). Examination of these gray-white contrasts provide a highly localized normalization of tissue values because the gray and white matter intensity from closely neighboring voxels would be expected to be similarly influenced by any imaging parameters due to the smoothness of the nuisance parameter maps (e.g., field B0/B1 inhomogeneities) which can be assumed to be smooth relative to the local normalization operation. Thus, presenting the intensity values as a ratio to bordering intensity values provides a unit that is normalized for the local imaging environment. The multilayer sampling procedure allows quantification of multiple contrast levels including contrast conservatively close to the gray/white border (20% gray matter/.5mm white matter) as well as more remote contrast such as outer gray matter to deep white matter (80% gray matter/1mm white matter). After calculation of the ratio features, values were smoothed with a Gaussian kernel of FWHM 5 mm for analysis utilizing a surface-based smoothing procedure that averages data across neighboring cortical locations. These microscale feature maps were integrated with the cortical thickness map for the full feature set (Fig. 1, panel d). Fig. 1 illustrates the flowchart of the MSSM procedure.

### 2.5 Multivariable analysis & AD classification

*Train-test split*. Data of AD and controls were randomly split into 80% training and 20% test sets. It is critical that train-test split is done at the very beginning of all the procedures before data preprocessing, feature extraction, and PLS. This allows the test set to be completely held out and never seen by any data processing or modeling. The training set was again split into training and validation sets, thereby having 60% training, 20% validation, and 20% test sets. The following procedures for feature extraction and model training were performed using the training and validation sets, and the test set was accessed strictly after the final model was established and trained.

*Feature selection*. The 8 gray/white matter contrasts and cortical thickness were used as primary features in the identification of individuals with AD and differentiation from matched controls.

*Vertex-wise PLS regression*. Each feature was normalized to have intensities between 0 and 1. Since we have more variables than observations and multicollinearity exists between the features, partial least squares (PLS) regression was used for dimensionality reduction for each vertex. The PLS method is used to find the direction in the feature space that explains the maximum variance direction in the diagnosis label space (i.e., AD or control). The reduced feature map (one component per vertex) was used to train classification models (Fig. 1, panel e).

*Model training*. Multiple commonly used machine learning models were trained independently and ensembled in the end for final decision-making. Models considered in the study are SVM with varying kernels (linear, sigmoid, polynomial, radial basis function), neural networks, random forest, logistic regression, k-nearest neighbors, and Gaussian process. Class labels were binarized and the probability of each class was estimated to enable analyses of receiver operating characteristic (ROC) and precision-recall (PR) curve. We trained these models while accounting for the class imbalance by giving each sample a weight inversely related to its class’s prevalence (class weights) in the training data. For linear models such as linear SVM and logistic regression, the loss function was modified by weighting the loss of each sample by its class weight. For tree-based algorithms, the class weights were used for reweighting the splitting criterion, (King et al., 2001, Pedregosa et al., 2011). The validation set was used to find hyperparameters that maximize the classification performance (Fig. 1, panel f).

*Ensemble learning*. We randomly initialized and trained each model 5 times and identified 3 models with the highest average performance in the validation set. The primary performance measure we used is the area under the ROC curve (AUROC). 4-fold cross-validation was used to measure the average AUROC for each weight initialization, thereby having 20 AUROC values to be averaged for each model. The top 3 models were ensembled by averaging the output of the models to form a single classifier, which makes the final decision (Fig. 1, panel g). Model ensembling is a process to combine the predictions of several learning algorithms and is known to yield performance better than a single model (Dietterich, 2000).

*Evaluation*. The inference was performed on the test set and its performance was reported—i.e., AUROC, the area under the PR curve (AUPRC), accuracy, sensitivity, and specificity. We also present ROC and PR curves. ROC curve displays a trade-off between the sensitivity and the specificity of the features over every possible decision threshold. PR curve, a less frequently used plot, on the other hand, displays the trade-off between precision (instead of specificity) and sensitivity (i.e., recall). PR curves can provide an accurate prediction of future classification performance due to the fact that they evaluate the fraction of true positives among positive predictions, therefore, can be more informative when applied to an imbalanced dataset in which the number of negatives outweighs the number of positives (Saito et al., 2015) (Fig. 1, panel h).

### 2.6 Prediction of progression from MCI to AD dementia

We examined whether the MSSM-based AD classification model trained above can be directly used to predict MCI progression to AD dementia to determine sensitivity to earlier pathology. The experiment used the same processing steps as the AD classification; however, the task was to distinguish MCI-C from the matched MCI-NC rather than AD from controls. In other words, the model trained on 80% of the AD/control data was tested on the MCI-C/MCI-NC data (Fig. 1, panel i). The evaluation procedure was similar to that used for AD classification.

### 2.7 Added value of MSSM

In order to examine the added value of the MSSM features over traditional morphometry measured by cortical thickness alone, the same procedure was performed with the cortical thickness map. In this case, the vertex-wise PLS regression was not needed. Note that not only cortical thickness but also MSSM has 1 feature per vertex since it went through dimensionality reduction with PLS. Effects of AD on MSSM and cortical thickness were analyzed using standard statistical contrast while correcting for multiple comparisons. Moreover, we identified regions where MSSM is more effective than cortical thickness in differentiating AD patients from cognitively intact matched controls using effect size analyses. To further validate the added clinical value of MSSM over cortical thickness, correlations were sought between the average MSSM value in the ‘extra’ regions and cognitive performance measured by FAQ within the AD group, in which ‘extra’ regions refer to regions where significant AD effects are found with MSSM but not with cortical thickness (CT). The correlations were analyzed in three different ways: 1) FAQ vs. mean MSSM extra; 2) FAQ vs. mean MSSM extra controlled for mean CT across the brain (y controlled for $x_{\mathrm{all}}$); 3) FAQ vs. mean MSSM extra controlled for mean CT across the significant regions (y controlled for $x_{\mathrm{sig}}$), where

$$y=\frac{1}{\left|MSSMextra\right|}\sum_{v\in MSSMextra}MSSM\left(v\right),$$

$$x_{\mathrm{all}}=\frac{1}{\left|CT_{\mathrm{all}}\right|}\sum_{v\in CT_{\mathrm{all}}}CT\left(v\right),$$

$$x_{\mathrm{sig}}=\frac{1}{\left|CT_{\mathrm{sig}}\right|}\sum_{v\in CT_{\mathrm{sig}}}CT\left(v\right),$$

and MSSMextra is a set of vertices where significant AD effects are found with MSSM but not with cortical thickness (CT), $CT_{\mathrm{all}}$ is a set of vertices throughout the cerebral cortex, $CT_{\mathrm{sig}}$ is a set of vertices where AD effects are significant in CT for each patient. In the classification studies, we compared the diagnostic (or prognostic) performance of MSSM with models using normalized hippocampal volume (normalized with estimated total intracranial volume (eTIV)) and models using cognitive performance (e.g., RAVLT Immediate and RAVLT Percent Forgetting, which are known to have a strong association with AD and progression from MCI to AD (Estévez-González et al., 2003, Wang et al., 2011, Gomar et al., 2014, Moradi et al., 2015)). Lastly, we examined whether adding demographic features—i.e., age, gender, education, race, and ethnicity—to MSSM helps in diagnostic performance.

## 3 Results

**Fig. 3:**
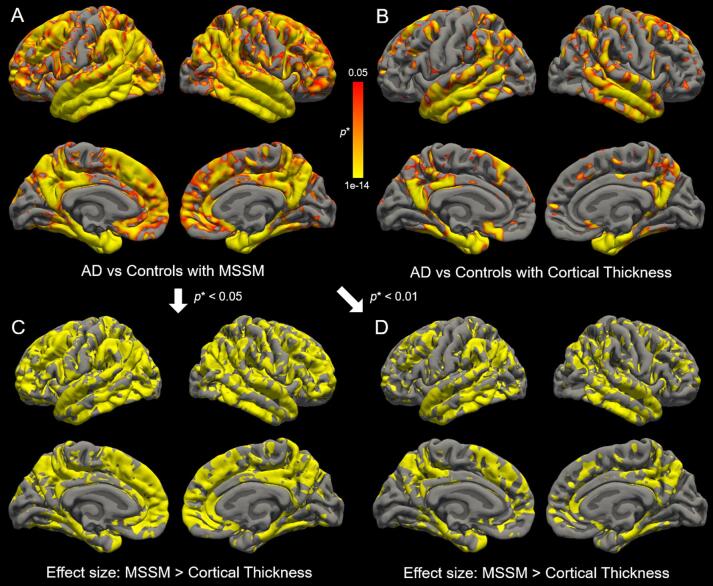
Effect of AD on MSSM and cortical thickness using standard statistical contrast. Use of the MSSM features doubled the number of vertices showing a statistical difference between AD patients and cognitively intact matched controls (A) compared to traditional cortical thickness measures (B) demonstrating the increased sensitivity of the MSSM metric. Note that not only cortical thickness but also MSSM has 1 feature per vertex since it went through dimensionality reduction with PLS. The colored regions represent vertices where the FDR-corrected *p*-value (p*) is lower than 0.05. The images with the threshold of 0.01 for A & B are available in Appendix. C & D show regions where MSSM is more effective than cortical thickness in differentiating AD patients from cognitively intact matched controls based on effect size analyses. The MSSM features were more effective compared to traditional cortical thickness measures in most regions, thus, only vertices showing a statistical difference between AD and controls with the MSSM features are shown at the p* < 0.05 level (C) and p* < 0.01 level (D). (For interpretation of the references to color in this figure legend, the reader is referred to the web version of this article.)

We first examined the added value of the MSSM features over traditional morphometry measured by cortical thickness alone in standard statistical group comparisons of AD compared to control participants. Use of the MSSM features doubled the number of significant vertices differentiating AD patients from controls compared to cortical thickness—71% of the total vertices significant for MSSM compared to 30% significant with cortical thickness using a threshold of p* < 0.05 (see Fig. 3). MSSM increased sensitivity to regions considered to degenerate in later stages of Alzheimer’s disease such as the frontal cortex.

The MSSM values in the ‘extra’ regions were significantly correlated with cognitive performance measured by FAQ, linearly (Pearson correlation) and/or monotonically (Spearman correlation; Table 2).

**Table 2:** Correlation between the average MSSM value in the ‘extra’ regions and cognitive performance measured by FAQ. The ‘extra’ regions refer to regions where significant AD effects are found with MSSM but not with cortical thickness (CT). The statistical significance was measured at the p* < 0.1 and p* < 0.05 levels.

	MSSM extra at p*<0.1		MSSM extra at p*<0.05	
	Pearson	Spearman	Pearson	Spearman
Mean MSSM extra (y)	*γ* = 0.33**	*ρ* = 0.33**	*γ* = 0.33**	*ρ* = 0.33**
Mean MSSM extra controlled for mean CT across the brain (y controlled for $x_{\mathrm{all}}$)	*γ* = 0.25*	*ρ* = 0.27*	*γ* = 0.25*	*ρ* = 0.26*
Mean MSSM extra controlled for mean CT across significant regions (y controlled for $x_{\mathrm{sig}}$)	*γ* = 0.24	*ρ* = 0.26*	*γ* = 0.23	*ρ* = 0.24*

* *p* < 0.05, ** *p* < 0.01.

We identified the most important feature at each vertex in the PLS analysis. Among the 9 features (8 GM/WM contrast features and cortical thickness), cortical thickness was the most important feature in 37% of the vertices and GM 20%/WM 0.5 mm in 31% of the vertices. It was followed by GM 20%/WM 1.0 mm, GM 40%/WM 0.5 mm, GM 80%/WM 0.5 mm, GM 40%/WM 1.0 mm, GM 60%/WM 0.5 mm, GM 80%/WM 1.0 mm, and GM 60%/WM 1.0 mm.

**Fig. 4:**
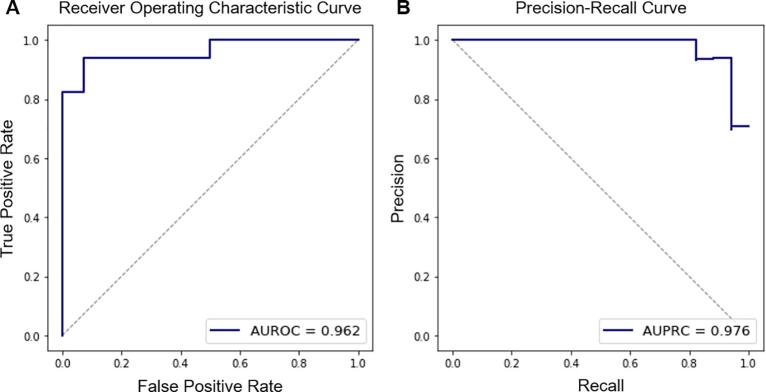
Receiver operating characteristic (ROC) curve (A) and precision-recall (PR) curve (B) in the prediction of AD patients using the MSSM features. They illustrate the model’s performance on the held-out test set. Performance metrics of the cortical thickness-based model are also shown. The ROC curve shows a trade-off between the sensitivity and the specificity; and the PR curve describes the trade-off between precision (i.e., positive predictive value) and recall (i.e., sensitivity) over all possible decision thresholds.

In the AD classification study, the MSSM features could differentiate AD from controls with an AUROC, AUPRC, accuracy, sensitivity, and specificity of 0.962, 0.976, 0.935, 0.941, and 0.929, respectively. The values were 0.920, 0.957, 0.903, 0.824, and 1.000 when cortical thickness was used, and 0.899, 0.926, 0.897, 0.938, and 0.846 when normalized hippocampal volume was used. The diagnostic values were also compared to neuropsychological values – i.e., RAVLT Immediate and RAVLT Percent Forgetting. No benefit was found when demographic features were added to the MSSM features. The diagnostic performance is summarized in Table 3. ROC curve and PR curve are shown in Fig. 4. Note that decision thresholds can be adjusted to increase either sensitivity or specificity at the cost of the other. The provided accuracy, sensitivity, and specificity in the table are based on a decision threshold chosen to minimize the number of false detection (false positives plus false negatives).

**Table 3:** Diagnostic accuracy for AD dementia.

Features	AUROC	AUPRC	Accuracy	Sensitivity	Specificity
RAVLT Immediate	0.947	0.962	0.903	0.824	1.000
RAVLT Percent Forgetting	0.893	0.931	0.871	0.824	0.929
Hippocampal Volume	0.899	0.926	0.897	0.938	0.846
Cortical Thickness	0.920	0.957	0.903	0.824	1.000
MSSM	0.962	0.976	0.935	0.941	0.929
MSSM + Demographic	0.962	0.976	0.935	0.941	0.929

**Fig. 5:**
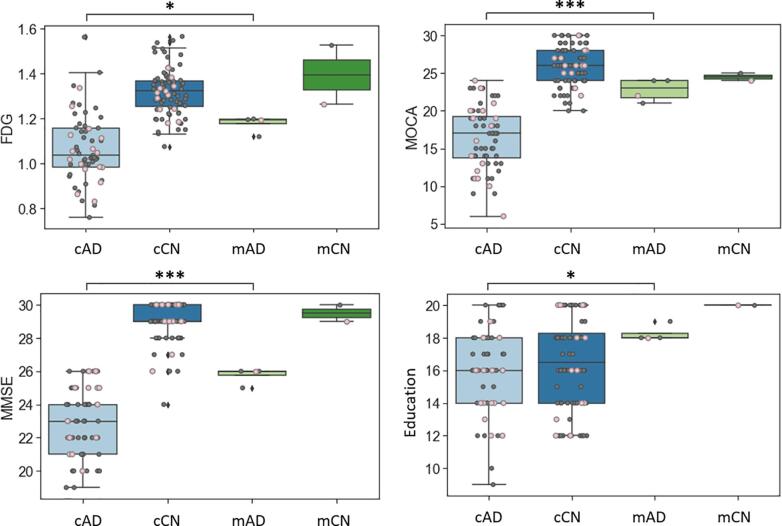
Clinical biomarkers of misclassified individuals in the detection of AD patients. Individuals in the test set are represented as pink circle and those in the training set are represented as dark-gray. cAD = correctly classified AD (true positive; ADNI diagnosis = AD; model prediction = AD), mAD = misclassified AD (false negative; ADNI diagnosis = AD; model prediction = Control), cCN = correctly classified control (true negative; ADNI diagnosis = Control; model prediction = Control), mCN = misclassified control (false positive; ADNI diagnosis = Control; model prediction = AD), * p < 0.05, *** p < 0.005.

**Fig. 6:**
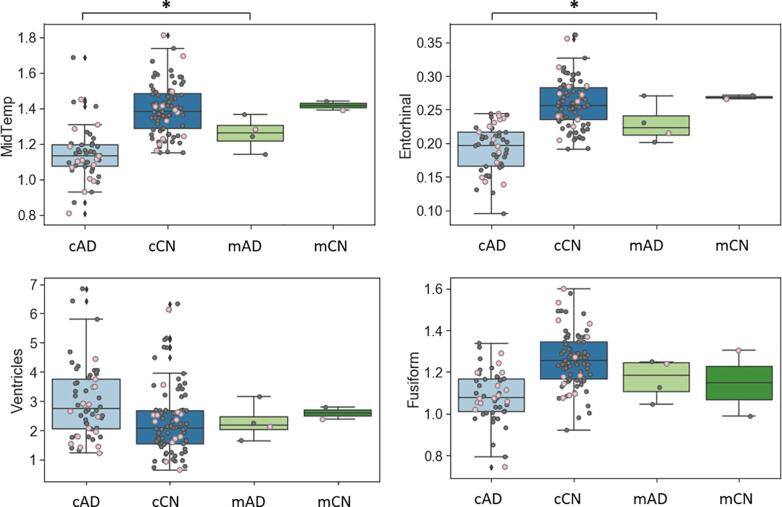
Structural biomarkers of misclassified individuals in the detection of AD patients. Volume of each brain region was corrected for estimated total intracranial volume (eTIV), presented as percentage of eTIV. Individuals in the test set are represented as pink circle and those in the training set are represented as dark-gray. cAD = correctly classified AD (true positive; ADNI diagnosis = AD; model prediction = AD), mAD = misclassified AD (false negative; ADNI diagnosis = AD; model prediction = Control), cCN = correctly classified control (true negative; ADNI diagnosis = Control; model prediction = Control), mCN = misclassified control (false positive; ADNI diagnosis = Control; model prediction = AD), * p < 0.05.

Post-hoc analyses of misclassified individuals revealed that the misclassified AD patients (mAD; false negative; ADNI diagnosis = AD, model prediction = control) were different from the median of correctly classified AD patients (cAD; true positive; ADNI diagnosis = AD, model prediction = AD) in multiple biomarkers (Fig. 5, Fig. 6). Statistical tests were not possible in the test set because there was only one mAD and one misclassified control (mCN; false positive; ADNI diagnosis = control, model prediction = AD). Hence, we included both training and testing data for these analyses. There were only two mCN, therefore, statistical tests (i.e., Mann Whitney *U* test) were performed only between mAD and AD. mAD had greater whole-brain standardized uptake value ratio (SUVR) for (Bloch and Friedrich, 2021) ^18^F-fluorodeoxyglucose (FDG) PET (*p* < 0.05), showed better performance on MoCA (*p* < 0.005) and MMSE (*p* < 0.005), and had higher education (*p* < 0.05) compared to cAD. Also, the volumes of middle temporal gyrus (*p* < 0.05) and entorhinal cortex (*p* < 0.05) in mAD were larger than cAD. Although not statistically tested due to the small sample size, the volumes of ventricles and fusiform gyrus in mCN were closer to cAD in their medians than the correctly classified controls (cCN; true negative; ADNI diagnosis = control, model prediction = control). The volume of each brain region was corrected for estimated total intracranial volume (eTIV), presented as a percentage of eTIV in Fig. 6.

**Fig. 7:**
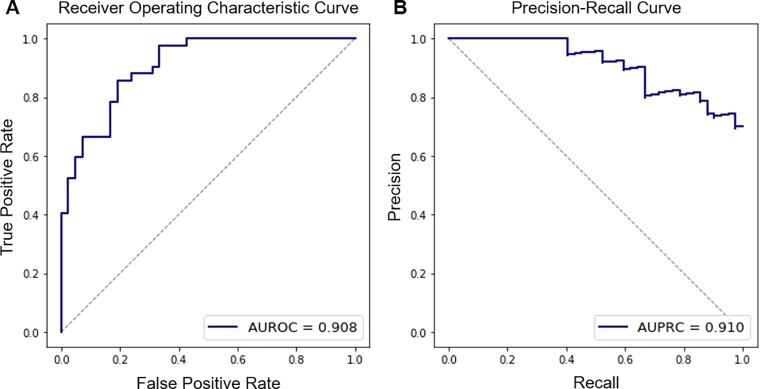
Receiver operating characteristic (ROC) curve (A) and precision-recall (PR) curve (B) in the prediction of MCI progression to AD dementia using the MSSM features. They illustrate the model’s performance on the held-out test set. Performance metrics of the cortical thickness-based model are also shown. The ROC curve shows a trade-off between the sensitivity and the specificity; and the PR curve describes the trade-off between precision (i.e., positive predictive value) and recall (i.e., sensitivity) over all possible decision thresholds.

In the MCI to AD progression prediction study, the MSSM features could differentiate MCI-C from MCI-NC with an AUROC, AUPRC, accuracy, sensitivity, and specificity of 0.908, 0.910, 0.833, 0.857, and 0.810, respectively. The values were 0.887, 0.893, 0.833, 0.857, and 0.810 when cortical thickness was used, and 0.834, 0.844, 0.797, 0.842, and 0.756 when the normalized hippocampal volume was used. The prognostic values were also compared to neuropsychological values – i.e., RAVLT Immediate and RAVLT Percent Forgetting. No benefit was found when demographic features were added to the MSSM features. The prognostic performance is summarized in Table 4. ROC curve and PR curve are shown in Fig. 7.

**Table 4:** Prognostic accuracy for AD progression from MCI.

Features	AUROC	AUPRC	Accuracy	Sensitivity	Specificity
RAVLT Immediate	0.853	0.845	0.810	0.857	0.762
RAVLT Percent Forgetting	0.767	0.714	0.762	0.762	0.762
Hippocampal Volume	0.834	0.844	0.797	0.842	0.756
Cortical Thickness	0.887	0.893	0.833	0.857	0.810
MSSM	0.908	0.910	0.833	0.857	0.810
MSSM + Demographic	0.908	0.910	0.833	0.857	0.810

**Fig. 8:**
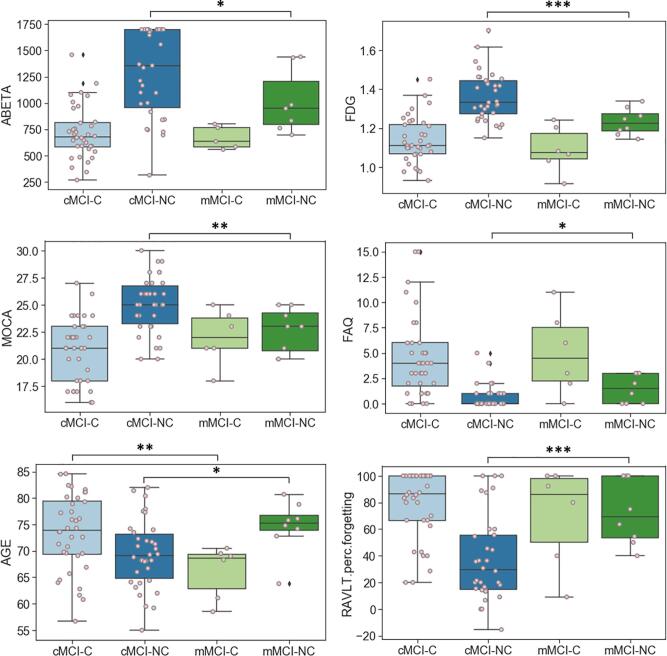
Clinical biomarkers of misclassified individuals in prediction of MCI to AD progression. CSF Aβ42 (ABETA) values were thresholded at 1700. cMCI-C = correctly classified MCI converters (true positive; ADNI diagnosis = MCI→AD; model prediction = MCI→AD), mMCI-C = misclassified MCI converters (false negative; ADNI diagnosis = MCI→AD; model prediction = MCI→MCI), cMCI-NC = correctly classified MCI non-converters (true negative; ADNI diagnosis = MCI→MCI; model prediction = MCI→MCI), mMCI-NC = misclassified MCI non-converters (false positive; ADNI diagnosis = MCI→MCI; model prediction = MCI→AD), * *p* < 0.05, ** *p* < 0.01, *** *p* < 0.005.

**Fig. 9:**
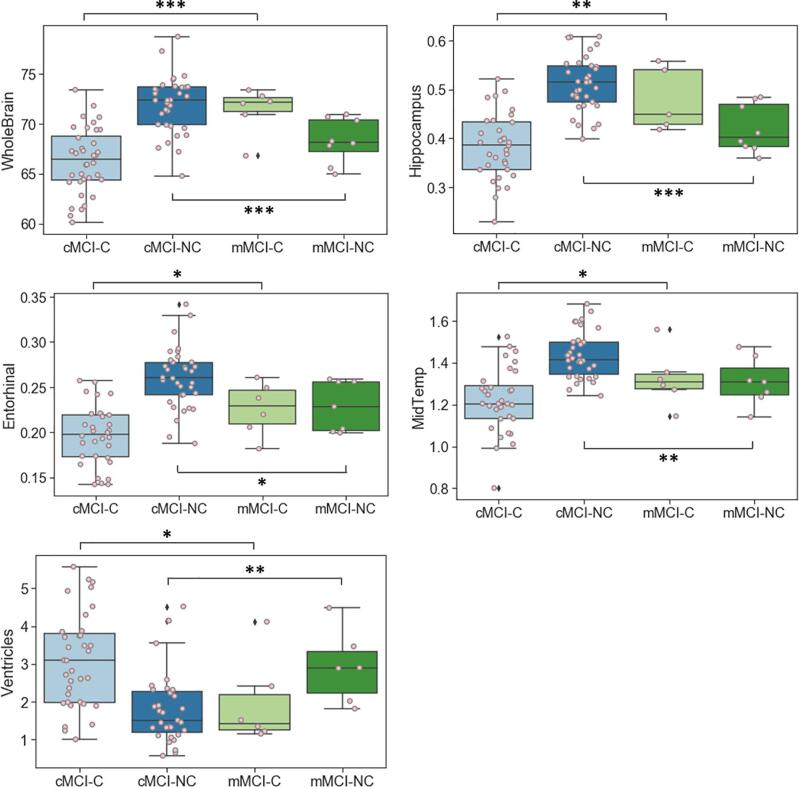
Structural biomarkers of misclassified individuals in the prediction of MCI to AD progression. Volume of each brain region was corrected for estimated total intracranial volume (eTIV), presented as percentage of eTIV. cMCI-C = correctly classified MCI converters (true positive; ADNI diagnosis = MCI→AD; model prediction = MCI→AD), mMCI-C = misclassified MCI converters (false negative; ADNI diagnosis = MCI→AD; model prediction = MCI→MCI), cMCI-NC = correctly classified MCI non-converters (true negative; ADNI diagnosis = MCI→MCI; model prediction = MCI→MCI), mMCI-NC = misclassified MCI non-converters (false positive; ADNI diagnosis = MCI→MCI; model prediction = MCI→AD), * *p* < 0.05, ** *p* < 0.01, *** *p* < 0.005.

Post-hoc analyses of misclassified individuals revealed that the misclassified MCI converters (mMCI-C; false negative; ADNI diagnosis = MCI→AD, model prediction = MCI→MCI) were different from the median of correctly classified MCI converters (cMCI-C; true positive; ADNI diagnosis = MCI→AD, model prediction = MCI→AD) in multiple biomarkers. mMCI-C were older (*p* < 0.05; Fig. 8) and had larger volumes of whole brain (*p* < 0.005), hippocampus (*p* < 0.005), entorhinal cortex (*p* < 0.05), and middle temporal gyrus (*p* < 0.05) compared with cMCI-C. mMCI-C had smaller volume of ventricles (*p* < 0.05; Fig. 9). The misclassified MCI non-converters (mMCI-NC; false positive; ADNI diagnosis = MCI→MCI, model prediction = MCI→AD) were different from the median of correctly classified MCI non-converters (cMCI-NC; true negative; ADNI diagnosis = MCI→MCI, model prediction = MCI→MCI) in various biomarkers. mMCI-NC were older (*p* < 0.05) and showed lower CSF Aβ42 (ABETA; *p* < 0.05) and FDG (*p* < 0.005) levels compared with cMCI-NC, but no statistical difference was found in tau. mMCI-NC showed worse performance on MoCA (*p* < 0.05), FAQ (*p* < 0.05), , and RAVLT Percent Forgetting (*p* < 0.005; Fig. 8). Also, structural markers were consistently worse in mMCI-NC—i.e., the volumes of the whole brain (*p* < 0.005), hippocampus (*p* < 0.005), entorhinal cortex (*p* < 0.05), middle temporal gyrus (*p* < 0.05), and ventricles (*p* < 0.05). The volume of each brain region was corrected for estimated total intracranial volume (eTIV), presented as percentage of eTIV in Fig. 9.

## 4 Discussion

We demonstrated that the proposed MSSM biomarkers from a standard T1-weighted image increase sensitivity to structural differences between AD patients and cognitively intact matched adults and can be used to detect patients with AD dementia and patients with MCI who progress to AD with a high degree of accuracy. Thus, this procedure may provide an optimal metric for the ‘N’ component of the ‘A-T-N’ biological framework for AD that is specific for signature patterns of AD neurodegeneration. Future applications can implement these procedures to additionally describe heterogeneity in atrophy patterns in ‘atypical’ AD patients. These measures can be used independently, or in a complementary manner to amyloid and tau biomarkers to complete the ‘A-T-N’ characterization of individual patients.

The current results show that there are alterations in tissue signal properties in AD patients that provide enhanced information about neurodegeneration relative to cortical thickness alone and are distinct from the effects of typical aging. Particularly strong effects were found in temporal and limbic areas, which is consistent with Salat et al. 2011 (Fischl and Dale, 2000) that used Open Access Series of Imaging Studies (OASIS) data (Dietterich, 2000). The study reported that the effects of AD on the gray matter to white matter contrast had unique variance relative to changes in cortical thickness alone and although there was substantial overlap between the effects, there was also regional differentiation between AD effects on thickness compared to intensity/contrast effects. Although cortical thickness component was most sensitive to group differences across the greatest percentage of vertices in our study, the gray 20%/white 0.5 mm contrast measure approached the percent vertices that cortical thickness achieved, and the vertices combined across all contrast metrics comprised a greater percentage of the total significant vertices than cortical thickness. The MSSM procedure that combines the two metrics exhibited substantially broader effects while cortical thickness showed more focal but stronger effects in a few areas. MSSM features more than doubled significant vertices statistically different in AD patients compared to controls relative to cortical thickness measures. The MSSM values in the ‘extra’ regions were significantly correlated with cognitive performance, which suggests that MSSM can measure AD-related lesions that are not captured by the standard morphometry. Regions most affected are those known to show early and aggressive degeneration from pathology studies48, (Dale et al., 1999). However, MSSM additionally highlighted later stage regions in the frontal cortex. This suggests that the tissue signal properties can be a microstructural marker of pathologic mechanisms that are more preserved from cortical atrophy or that have a distinct longitudinal pattern in the disease process.

As a ‘proof of concept’, we used the MSSM features to differentiate AD patients from cognitively healthy matched controls and it revealed competitive diagnostic performance (AUROC = 0.962, AUPRC = 0.976), which is comparable to but slightly higher than our cortical thickness-based model that used similar procedures and models based on hippocampal volume or RAVLT cognitive test scores. It also outperformed models that used one or more MRI features of cortical thickness, hippocampal volume, shape, texture, WM hyperintensity, and volumetrics, (Cho et al., 2012, Wolz et al., 2011, Sørensen et al., 2016, Sørensen et al., 2017, McEvoy et al., 2009), MRI-based convolutional neural network (CNN) models, (Lu et al., 2021), and PET-based models, (Mattsson et al., 2019, Janghel and Rathore, 2021), and a model combining cortical thickness and default mode network functional connectivity (Park et al., 2017) even though one-to-one comparisons are not possible. Higher performance was reported in studies including the ones that combined MRI and PET (Suk et al., 2014), used functional MRI data, (Mattsson et al., 2019), and combined MRI with genotype data and cognitive performance (Bloch and Friedrich, 2021). Demographic features to MSSM did not enhance performance in this sample, which implies that the MSSM features capture the variance of the demographic features in most part or that classification performance is ceiling.

We also used the MSSM features to predict MCI patients who progress to AD dementia within 3 years and it showed prominent prognostic performance (AUROC = 0.908, AURPC = 0.910), which is comparable to but slightly higher than the cortical thickness-based model that used similar procedures and models based on hippocampal volume or RAVLT cognitive test scores. It also outperformed models that use one or more MRI features of cortical thickness, hippocampal volume, and volumetric features, (Sørensen et al., 2016, Popuri et al., 2020, Zhu et al., 2017), MRI-based CNN models, (Gao et al., 2020, Lu et al., 2018), a model that combines MRI, genotypes, and gene expression profiles (Li et al., 2021), a model combining MRI morphometrics, CSF and cognitive measures (Ye et al., 2012), and models using PET or PET combined with MRI, (Liu et al., 2014, Lu et al., 2018, Zhu et al., 2017). Higher performance was reported in a few studies that used longitudinal MRI, (Sun et al., 2017) and that combined MRI with cognitive performance (Tong et al., 2017). This work demonstrates the possibility to use this technique, which requires only a single T1-weighted MRI, in diagnostic support clinically, as well as to screen individuals who are likely to be a candidate for more intensive biomarker assessment. Future work will apply the MSSM procedures in individuals with earlier impairment and/or pathology stages, MCI with conversion to other types of dementia, and preclinical at-risk individuals (Li et al., 2021) to determine the benefits of these novel features.

The analyses of the misclassified individuals in the AD classification study showed that the misclassified AD participants (mAD)—i.e., ADNI diagnosis = AD; model prediction = Control—looked more like controls than the correctly-classified AD (cAD) in multiple biomarkers (Fig. 5, Fig. 6). It may suggest that misclassification of AD can be due to clinical mislabeling of AD patients with etiologies other than AD or AD atrophy variants. The former hypothesis is less likely given that mAD showed positive amyloid PET. Given that the prediction models are created from group maps, any AD that is not classified correctly must by definition have a substantially different pattern of degeneration than is typical for the group. Further investigation is needed to analyze individual cortical atrophy patterns for mAD. The use of the MSSM biomarkers showed a 12%-point increase in sensitivity but a 7%-point decrease in specificity based on the chosen decision thresholds, therefore, it is difficult to argue that the proposed methods are always preferred. However, it is critical to note that performance is based on clinical labels, and these labels may include misdiagnosis (Belathur Suresh et al., 2018). There was one misclassified control (mCN) in the training set and test set each. The volume of their ventricles and fusiform gyrus were closer to cAD than the correctly-classified controls (cCN); however, it was difficult to explain the cause of the false positives with other biomarkers (Fig. 5, Fig. 6), thus it may suggest a need for further model optimization to improve specificity. Alternatively, it is possible that MSSM is overly sensitive to pathology generically (including non-AD pathologies). We are interested in exploring this concept further with more data in future work.

In the MCI to AD progression study, we observed that the misclassified MCI converters (mMCI-C) were younger, had better brain based on volumetrics, and better learning performance than the correctly-classified MCI converters (cMCI-C; Fig. 8, Fig. 9). This is potentially suggestive of conversion due to other factors. For example, an mMCI-C individual with the smallest ventricular volume had significantly enlarged perivascular spaces that seemed atypical. The misclassified MCI non-converters (mMCI-NC) were older, had worse brain based on volumetrics, worse cognition, and higher CSF amyloid level than the correctly-classified MCI non-converters (cMCI-NC; Fig. 8, Fig. 9). This may suggest that they had early AD, but are protected in some way such as mechanisms of resilience against the effects of AD Pathology. Note that the misclassifications in both experiments were very low and these interpretations are speculative and need further investigation with data of more participants.

Additional studies are necessary to determine the optimal application of the MSSM procedure. Although we trained and evaluated the model using the data acquired at multiple (33) imaging sites that use different scanner models, we limited this initial investigation to Siemens scanners only. Thus, in the current form, the procedures may be best applied to cohorts that have been scanned using a single protocol. We briefly note, however, that performance classifying GE data with the Siemens-trained MSSM model was not markedly compromised and outperformed the Siemens-trained cortical thickness model in our preliminary analyses. We plan to put significant effort into this procedure to assure maximal performance across vendors, hence, do not provide this information in this paper except to note that we believe that this procedure is generalizable. Future studies will explicitly include cross-vendor information into the MSSM preprocessing and statistical models to enhance generalizability across manufacturers and cohorts. The MSSM features may provide an indirect index of tissue microstructure but can be impacted by other imaging aspects and we will plan to validate these measures against gold standard microstructural measurements. Future studies will add more macrostructural features such as mean curvature, surface area, and gyrification index. Also, additional studies are needed to include participants of more diverse race/ethnicity as well as greater disease heterogeneity, both in stage as well as type. With these caveats, we conclude that the MSSM procedure is more sensitive for the detection of AD neurodegeneration and is preferable to morphometry alone.

## Declaration of Competing Interest

In the past 36 months, outside the submitted work, B.C.D. has received research support from NIH; consulted for Acadia, Alector, Arkuda, Biogen, Denali, Lilly, Merck, Novartis, Takeda, and Wave LifeSciences; performed editorial duties with payment for Elsevier (Neuroimage: Clinical and Cortex); and received royalties from Oxford University Press and Cambridge University Press. D.H.S. has held leadership or fiduciary role in Niji Corp, Smart Ion, and Salat Research Consulting. J.M.R. received a networking travel grant from the Dutch government. The other authors report no declarations of interest.
