# Supplementary material for: Multiscale structural mapping of Alzheimer’s disease neurodegeneration
Source: Neuroimage Clin. 2022 Jan 22;33:102948. doi: 10.1016/j.nicl.2022.102948 (PMC8814667; doi:10.1016/j.nicl.2022.102948)
Supplement: Supplementary data 1 [file mmc1.docx]

**Inclusion criteria**. The ADNI diagnosis criteria were made based on the National Institute of Neurological Disorders and Stroke and the Alzheimer’s Disease and Related Disorders Association^1^. The inclusion criteria for AD were an absence of significant impairment in cognitive functions or activities of daily living, Clinical Dementia Rating (CDR) of 0, free of memory complaints, and haven’t been diagnosed for AD or MCI. The inclusion criteria for MCI were the presence of memory complaints and abnormal memory function, CDR score of 0.5, mini-mental state exam (MMSE) score between 24 and 30 (inclusive), and general cognition and functional performance sufficiently preserved such that a diagnosis of AD cannot be made at the time of the screening visit.

1. McKhann G, Drachman D, Folstein M, Katzman R, Price D, Stadlan EM. Clinical diagnosis of Alzheimer’s disease: Report of the NINCDS-ADRDA Work Group under the auspices of Department of Health and Human Services Task Force on Alzheimer’s Disease. Neurology. 1984;34(7):939-939.


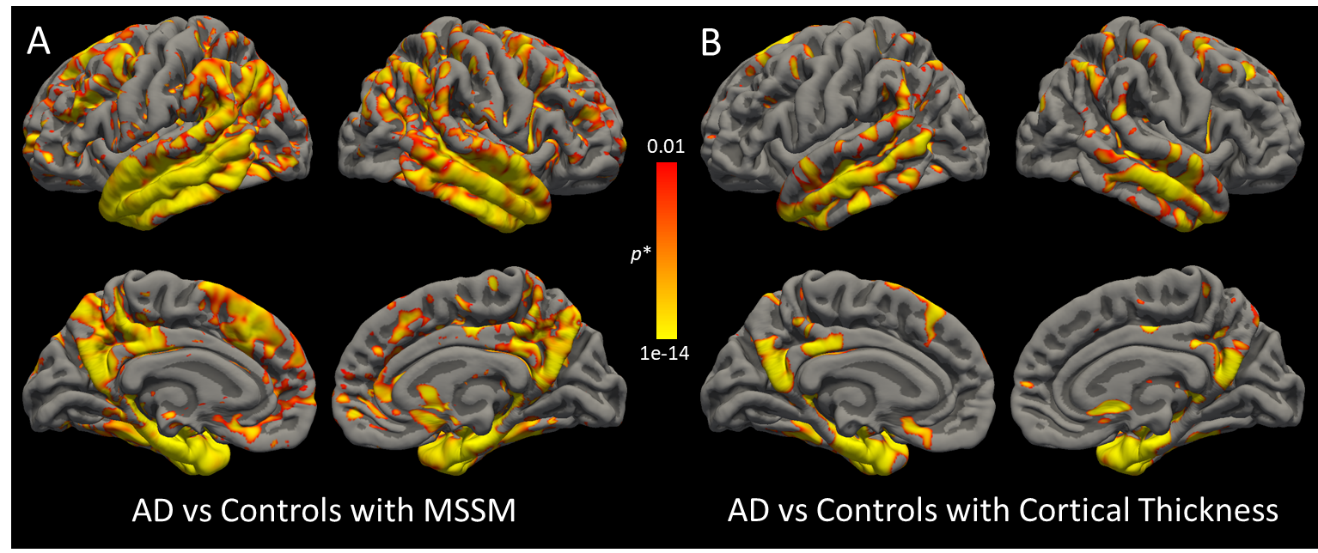


**Figure A.1.** Effect of AD on MSSM and cortical thickness using standard statistical contrast. Use of the MSSM features doubled the number of vertices showing a statistical difference between AD patients and cognitively intact matched controls (A) compared to traditional cortical thickness measures (B) demonstrating the increased sensitivity of the MSSM metric. Note that not only cortical thickness but also MSSM has 1 feature per vertex since it went through dimensionality reduction with PLS. The colored regions represent vertices where the FDR-corrected *p*-value (*p**) is lower than 0.01.
